# ELOVL4 Mutations That Cause Spinocerebellar Ataxia-34 Differentially Alter Very Long Chain Fatty Acid Biosynthesis

**DOI:** 10.1016/j.jlr.2022.100317

**Published:** 2022-12-01

**Authors:** Yeboah Kofi Gyening, Neeraj Kumar Chauhan, Madison Tytanic, Vicki Ea, Richard S. Brush, Martin-Paul Agbaga

**Affiliations:** 1 Departments of Cell Biology, University of Oklahoma Health Sciences Center, Oklahoma City, Oklahoma, USA; 2 Department of Ophthalmology, Dean McGee Eye Institute, Oklahoma City, Oklahoma, USA; 3 Departments of Ophthalmology, University of Oklahoma Health Sciences Center, Oklahoma City, Oklahoma, USA

**Keywords:** lipids, elongation of very long chain fatty acid-4, eye/retina, saturated fatty acid, fatty acid metabolism, omega-3 fatty acids, very long chain polyunsaturated fatty acids, autosomal dominant Stargardt macular dystrophy, tissue-specific pathologies, erythrokeratodermia variabilis, EKV, Erythrokeratodermia variabilis, ELOVL, elongation of very long-chain fatty acid, FAME, FA methyl ester, SCA, spinocerebellar ataxia, UT, untransduced, VLC, very long chain, VLC-SFA, very long chain saturated FA

## Abstract

The FA Elongase-4 (ELOVL4) enzyme mediates biosynthesis of both very long chain (VLC)-PUFAs and VLC-saturated FA (VLC-SFAs). VLC-PUFAs play critical roles in retina and sperm function, whereas VLC-SFAs are predominantly associated with brain function and maintenance of the skin permeability barrier. While some ELOVL4 mutations cause Autosomal Dominant Stargardt-like Macular Dystrophy (STGD3), other ELOVL4 point mutations, such as L168F and W246G, affect the brain and/or skin, leading to Spinocerebellar Ataxia-34 (SCA34) and Erythrokeratodermia variabilis. The mechanisms by which these ELOVL4 mutations alter VLC-PUFA and VLC-SFA biosynthesis to cause the different tissue-specific pathologies are not well understood. To understand how these mutations alter VLC-PUFA and VLC-SFA biosynthesis, we expressed WT-ELOVL4, L168F, and W246G ELOVL4 variants in cell culture and supplemented the cultures with VLC-PUFA or VLC-SFA precursors. Total lipids were extracted, converted to FA methyl esters, and quantified by gas chromatography. We showed that L168F and W246G mutants were capable of VLC-PUFA biosynthesis. W246G synthesized and accumulated 32:6n3, while L168F exhibited gain of function in VLC-PUFA biosynthesis as it made 38:5n3, which we did not detect in WT-ELOVL4 or W246G-expressing cells. However, compared with WT-ELOVL4, both L168F and W246G mutants were deficient in VLC-SFA biosynthesis, especially the W246G protein, which showed negligible VLC-SFA biosynthesis. These results suggest VLC-PUFA biosynthetic capabilities of L168F and W246G in the retina, which may explain the lack of retinal phenotype in SCA34. Defects in VLC-SFA biosynthesis by these variants may be a contributing factor to the pathogenic mechanism of SCA34 and Erythrokeratodermia variabilis.

FAs are essential for cell signaling, production of energy, and modulation of membrane fluidity as critical components of cell membranes. As a result, deficiency in certain FAs is detrimental to normal cellular health and causes many human diseases (1, 2). Previous studies clearly demonstrate the essential role of PUFAs, particularly 18:2n6 and 18:3n3, and their elongated n6 and n3 derivatives in human diseases (1, 3, 4). Elongation of very long-chain fatty acid 4 (ELOVL4), a member of a large family of FA elongases, mediates tissue-specific biosynthesis of both very long chain (VLC)-PUFA and very long chain-saturated FA (VLC-SFA) that we collectively call VLC-FAs (≥28 carbon atoms) (5, 6, 7, 8). VLC-FA, found in the retina, brain, Meibomian glands, skin, and sperm, play critical roles in neuroprotection, skin permeability barrier maintenance, and sperm function (7, 8, 9, 10, 11, 12, 13, 14, 15, 16, 17). Different mutations in *ELOVL*4 have been reported to cause a number of tissue-specific disorders in humans. However, the mechanism by which ELOVL4 and its VLC-FA products mediate their tissue-specific functions is not fully understood.

In the retina, depletion of VLC-PUFAs that are mostly enriched in phosphatidylcholine leads to a significant decline in visual function (9, 17, 18). For instance, conditional deletion of *Elovl4* in photoreceptor cells in mice leads to a significant decline in VLC-PUFAs, resulting in a significant reduction in rod responses and loss of rod photoreceptor nuclei compared with their wild type (WT) littermates (9). Additionally, studies from cell culture and animal models of STGD3 demonstrate that the STGD3 mutant protein’s inability to synthesize VLC-PUFA may be a primary contributing factor to the pathology (17, 18, 19). VLC-PUFA may also aid in phototransduction, as it is shown to be tightly associated with rhodopsin (20, 21, 22, 23).

In the brain and skin, ELOVL4 makes VLC-SFAs that are incorporated into complex sphingolipids that are necessary for proper brain and skin health. Depletion of skin VLC-SFA in mice homozygous for the STGD3 alleles and mice with global knockout of *Elovl4* leads to the loss of ω-O-acyl ceramides, which are key in maintaining the hydrophobic components of the skin (8, 17, 24, 25). These animals, therefore, develop scaly, wrinkled skin, compromised epidermal permeability barrier function, and die within a few hours after birth (8, 17, 24, 25). Expressing WT *Elovl4* using skin-specific promoters rescues these mice from neonatal lethality (7, 15). However, these skin-rescued mice develop seizures and die by postnatal day 21 due to the lack of VLC-FA products in the brain (7), which underscores the critical role of ELOVL4 and its VLC-FA products for survival. Furthermore, VLC-SFAs, primarily 28:0 and 30:0 SFAs enriched specifically in synaptic vesicles prepared from baboon hippocampus, are shown to regulate presynaptic transmitter release kinetics in cultured mouse hippocampal neurons (7). We showed that increased presynaptic release rates measured in the hippocampal neurons from homozygous *Elovl4*^*Stgd3/Stgd3*^ mice could be rescued to normal WT levels by supplementing primary hippocampal neuronal cultures with 28:0 and 30:0 VLC-SFA (7).

Since the first report of STGD3 causing *ELOVL4* mutations (26, 27), numerous mutations in *ELOVL4* have been discovered and reported. While some of the *ELOVL4* mutations in humans are reported to only affect the retina and cause STGD3 without any other neurological or skin pathologies, other mutations affect the brain and/or skin, leading to spinocerebellar ataxia 34 (SCA34) and Erythrokeratodermia variabilis (EKV) without any reported retina degeneration (16, 26, 28, 29, 30, 31, 32, 33, 34, 35). EKV represents a group of genetic skin disorders characterized by reddened, dry, and thickened skin, while SCA is characterized by severe degenerative changes in the cerebellum leading to motor deficits and cognitive decline (36, 37). The first reported heterozygous *ELOVL4* mutation to cause SCA34 with an average onset of disease at 51 years of age was reported in a French-Canadian family bearing a transversion mutation, c.540G > C (p. L168F), in exon 4 of *ELOVL4* (16). Affected family members reported a skin phenotype consisting of EKV and atrophy of the cerebellum and the pons (30). The following year, another SCA34-causing mutation, c.736T > G (p. W246G) in exon 6 of *ELOVL4,* was discovered in a Japanese family who developed progressive ataxia and atrophy of the pons with an average onset of disease at 34 years of age (31). Other SCA34-causing mutations, c.539A > C (p. G180P), c.512T > C, (p. I171T), and c.698C > T (p. T233M), have been reported with or without the presence of EKV (32, 33, 34).

It is therefore becoming clear that ELOVL4 and its VLC-FA products are critical to health. However, how the different mutations in *ELOVL4* trigger distinct tissue-specific human disorders that include blindness, age-related cerebellar atrophy, ataxia, and skin disorders is a puzzling question that remains to be answered. In addition, how VLC-PUFA and VLC-SFA are differentially synthesized and incorporated into different lipid classes in different tissues and how mechanisms by which the different ELOVL4 mutations affect these critical factors to cause different tissue-specific diseases are not completely known. We therefore seek to understand how the different *ELOVL4* mutations alter VLC-SFA and VLC-PUFA biosynthesis to cause different tissue-specific disorders.

Based on the tissue-specific distribution of VLC-FA, with VLC-PUFA enriched in the retina and VLC-SFA predominantly in the brain and skin (7, 8, 10, 17), patients with SCA34 pathology without any reported retinal degeneration, as in those carrying the L168F and W246G ELOVL4 mutations (30, 31), may have an alteration in the quantities and types of VLC-SFAs produced. To determine how the levels of VLC-PUFA and VLC-SFA species may be altered in SCA34 disease, we utilized two ELOVL4 mutations, L168F and W246G, which cause SCA34 with different ages of onset of disease and without any reported retinal degeneration in our experiments. We developed in vitro assays and demonstrated that the two ELOVL4 mutations, which cause age-related cerebellar atrophy in SCA34 and EKV in humans, have the biosynthetic ability to make VLC-PUFA. Interestingly, we found that W246G made higher amounts of 32:6n3 VLC-PUFA but was defective in further elongation of the C32 FAs. The L168F protein seemed to exhibit a gain of function in VLC-PUFA biosynthesis, as it made 38:5n3 VLC-PUFA that we did not detect in either the WT-ELOVL4 or the W246G-expressing cells. However, these mutant proteins were deficient in making VLC-SFAs, especially the W246G protein, which showed negligible VLC-SFA biosynthesis activity in vitro. Our results may explain how the different ELOVL4 mutations differentially affect VLC-PUFA relative to VLC-SFA biosynthesis to cause different tissue-specific disorders in humans.

## Materials and Methods

### Cell culture

ARPE19 cells (ATCC, Manassas, VA) were cultured in DMEM-nutrient mixture F-12 (Invitrogen, Waltham, MA) supplemented with 10% heat-inactivated FBS (Sigma-Aldrich, St. Louis, MO) and 100 μg/ml of normocin antibiotics (Invivogen, San Diego, CA). HEK293T cells (ATCC, Manassas, VA) were grown in DMEM medium supplemented with 10% FBS and antibiotics. HEK293 cells were transduced with 4.695 × 10^8^ infectious forming units per ml of adenovirus for the experiments.

### Animals

We used the CRISPR/Cas9 gene editing method to generate a heterozygous Long-Evans rat model of the human SCA34 by knock-in of the c.736T>G, p.W246G mutation in ELOVL4 that causes human SCA34 (31) . Further details of the generation of the knock-in rat line are previously described (14, 38). All animal experiments were performed using retinas from 12-month-old wild type (WT), heterozygous (HET), and homozygous W246G mutant (MUT) Long-Evans rats, with genotype confirmed by PCR. Rats were maintained in a pathogen-free barrier facility on a 12-h light:12-h dark daily light cycle (∼25–40 lux at cage level) with food and water available at all times. All animal procedures were approved by the Institutional Animal Care and Use Committee of the University of Oklahoma Health Sciences Center and conformed to the National Institute of Health Guide for the Care and Use of Laboratory Animals, US Public Health Service guidelines and the Association for Research in Vision and Ophthalmology Resolution on the Use of Animals in Research.

### Adenoviral infection

Recombinant adenoviruses for the overexpression of WT ELOVL4, L168F ELOVL4, W246G ELOVL4, and GFP were designed and viral particles were generated by Vector Builder (Vector Builder, Chicago, IL). All viruses were amplified into high-titer stocks through the propagation in HEK293 cells, purified by the cesium chloride method as previously described (5, 6, 19), and dialyzed in buffer containing 10 mM Tris-HCl, pH 8.0, 80 mM NaCl, 2 mM MgCl_2_ buffer, and 10% glycerol. Infectious adenovirus titer was determined using the Adeno-X rapid titer kit (Takara, San Jose, CA), and infectivity was expressed as infectious forming units per ml.

### Western blot analyses

HEK293 cell pellets or whole retinas were lysed in a lysis buffer containing 1% (w/v) SDS, 10 mM Tris, and 1 mM EDTA, pH 8.0. Following sonication, lysates were cleared by centrifugation at 18,000 *g* for 10 min and protein concentration was determined by the Pierce BCA assay (Thermo Fisher Scientific, Rockford, IL). Equivalent amounts of protein samples were separated on 12% SDS-PAGE and transferred onto nitrocellulose membranes. Membranes were blocked with 5% nonfat dry milk (Bio-Rad, Hercules, CA) and incubated with primary antibody diluted in 3% milk overnight, followed by horseradish peroxidase-conjugated secondary goat anti-mouse or donkey anti-rabbit IgG for 1 h at room temperature. Immunoreactivity was detected by chemiluminescence using Super-Signal West Pico Plus Sensitivity Substrate (Thermo Fisher Scientific, Rockford, IL). Membranes were reprobed as necessary for the various markers. Primary antibodies used were as follows: anti-ELOVL4 at 1:1,000 (5), anti-MYC #2276 at 1:5,000 (Cell Signaling Technology, Waltham, MA), anti-HA#2367 at 1:2,000 (Cell Signaling Technology, Waltham, MA), anti-β-Tubulin #66240-1 at 1:10,000 (Proteintech, Rosemont, IL), and anti-GFP # ab290 at 1:3,000 (Abcam, Waltham, MA).

### Subcellular protein fractionation

The subcellular protein fractionation kit (Thermo Fisher Scientific, Rockford, IL) was used to fractionate proteins into the membrane and nuclear and cytoplasmic fractions from cultured ARPE-19 cells transduced with the Myc-tagged *Elovl4* variants for 48 h. According to the manufacturer’s instructions, the supernatants obtained from the membrane, cytoplasmic, and nuclear fractions were separated on 12% gels by SDS-PAGE and analyzed by Western blotting after the protein concentration was determined by the BCA method. Primary antibodies used were anti-MEK1/2 # D1A5 at 1:2,000 (Cell Signaling Technology, Danvers, MA), anti-CALNEXIN # 2679 at 1:2,000 (Cell Signaling Technology, Danvers, MA), anti-HISTONE 3 #D1H2 at 1:1,000 (Cell Signaling Technology, Danvers, MA), and anti-MYC #2276 (Cell Signaling Technology, Danvers, MA).

### Immunocytochemistry

ARPE-19 cells were grown on gelatin-coated coverslips and transduced with WT *Elovl4*, L168F, and W246G *Elovl4*. After 48 h, transduced cells were rinsed with 0.1 M PBS (pH 7.5) and fixed with prechilled 100% methanol for 10 min. Following fixation, cells were washed three times with 0.1 M PBS. Cells were then blocked and permeabilized with 5% goat serum with 0.05% Triton-X diluted in PBS for 1 h. Coverslips were incubated with primary rabbit anti-MYC antibody and anti-CALNEXIN antibody at 1:200 (Cell Signaling Technology, Danvers, MA) overnight at 4 °C. The next day, cells were washed and incubated with secondary antirabbit conjugated with Alexa Fluor 488 dye at 1:200 (Thermo Fisher Scientific, Rockford, IL) and anti-mouse conjugated with Alexa Fluor 568 dye at 1:200 (Thermo Fisher Scientific, Rockford, IL). The coverslips were then washed and mounted with Vectashield with 4',6-diamidino-2-phenylindole mounting medium (Vector Labs, Newark, CA) and imaged by confocal microscopy.

### FA treatment

HEK293 cells (7.5 × 10^6^) were grown in a 10 cm^2^ tissue culture dish and treated with media containing the FA precursors. Sodium salts of the FAs were conjugated with freshly made fraction V FA-free BSA (Sigma, St. Louis, MO) in a ratio of 4:1 FA: BSA for 20:5n3 (EPA U-99-A) (Nuchek, Elysian, MN), 24:0 (lignoceric acid, N-24-A) ( Nuchek, Elysian, MN), and the VLC-PUFA 34:5n3 (BASF Pharma, Florham Park, NJ). Cells were treated with 30 μg/ml of the fatty acid in media for 72 h. Following treatment, cells were harvested and washed once in 0.1 M PBS containing 50 μM of fraction V FA-free BSA (Sigma, St Louis, MO) to sequester excess free FA, followed by an additional wash with PBS only. The cell pellets were stored at −80 °C until further processing for lipid analysis.

### Lipid extraction and analysis

Total lipids were extracted from cells from sample homogenate equivalent to 2.0 mg of protein and retina according to the procedure described by Bligh and Dyer (39). To each lipid extract were added 25 nmol of 15:0 and 17:0 as internal standards. FA methyl esters (FAMEs) were generated by acid methanolysis with 16% HCl in methanol overnight at 85 °C. Following methanolysis, FAMEs were extracted into hexane and isolated by TLC using 80:20 hexane:ether (v/v) mobile phase. The plate was stained with 2,7-dichlorofluorescein and the FAMEs were recovered and extracted into hexane. The FAMEs extract was dried under nitrogen and resuspended in 20 μl of nonane and analyzed by GC-MS and GC-Flame ionization detection (6, 19).

### Statistical analysis

Statistical differences between experimental groups were analyzed using ANOVA with Tukey’s post hoc test and Šídák’s post hoc test using GraphPad Prism 9 software (GraphPad, La Jolla, CA). Data are represented as the mean ± SD. Significance is indicated by *P* value measurements, with a *P* < 0.05 considered significant; ∗*P* < 0.05; ∗∗*P* < 0.01; ∗∗∗*P* < 0.001; ∗∗∗∗*P* < 0.0001.

## Results

### Expression and subcellular localization of W246G and L168F ELOVL4

**Fig. 1:**
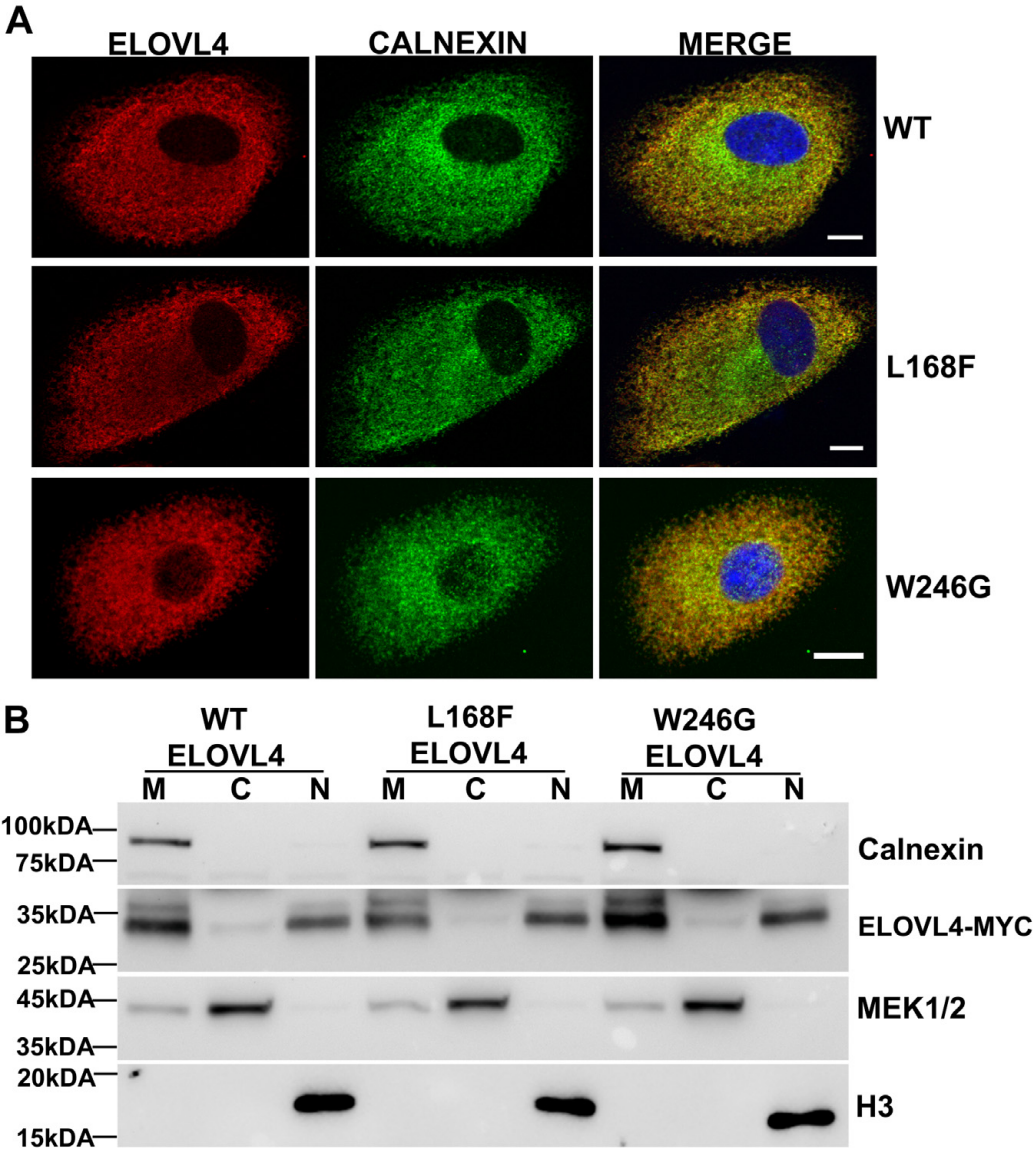
WT ELOVL4 and ELOVL4 SCA34 mutants L168F and W246G localize in the ER. A: ARPE-19 cells transduced with MYC-ELOVL4 constructs were immunostained for MYC (red) and CALNEXIN (green). WT, L168F, and W246G colocalized with ER transmembrane marker Calnexin. (Scale bar, 10 μm). B: Localization of WT ELOVL4 and SCA34 L168F and W246G mutant proteins by subcellular fractionation. Equal amounts of protein from membrane, cytoplasmic, and nuclear fractions of ARPE-19 cells transduced with MYC-ELOVL4 constructs were subjected to Western blot analysis using MYC antibody. CALNEXIN was used as a marker for the membrane (M), MEK1/2 as a loading control for the cytoplasmic fraction (C), and Histone 3 for the nuclear fraction (N). ELOVL, elongation of very long-chain fatty acid; SCA, spinocerebrellar ataxia.

The critical function of ELOVL4 in the biosynthesis of VLC-FA is dependent on its localization to the ER and its histidine catalytic core (6, 19). Based on recently predicted topology, the W246G ELOVL4 mutation is located at the border between the luminal loop in the ER lumen and the 7th transmembrane region (31). The L168F mutation, on the other hand, is 6 amino acid residues away from the histidine catalytic core of ELOVL4 located in the 4th transmembrane region (30). Unlike the 5-base pair deletion STGD3-causing ELOVL4 mutation that results in expressing a truncated protein losing the ER retention/retrieval motif, all SCA34-causing ELOVL4 mutations are point mutations that result in expression of the full-length mutant ELOVL4 proteins possessing the essential ER retention/retrieval motif and histidine catalytic core that are crucial for VLC-FA biosynthesis (6). To explore the impact of the W246G and L168F mutations on ELOVL4 subcellular localization and VLC-FA biosynthesis, we generated adenoviral particles of Myc-tagged WT mouse *Elovl4*, Myc-tagged W246G mouse *Elovl4*, and Myc-tagged L168F mouse *Elovl4*. We determined the subcellular localization of the W246G and L168F mutant proteins by immunofluorescence microscopy after transducing ARPE-19 cells with the different viral particles as previously described (19). Immunofluorescence studies showed that WT, L168F, and W246G colocalized with the ER marker calnexin, indicating their proper localization in the ER (Fig. 1A). To further confirm the ER localization of the WT, L168F, and W246G ELOVL4 variants in the transduced ARPE-19 cells, we fractionated the transduced cells into the cytoplasmic, membrane, and nuclear-enriched fractions and analyzed ELOVL4 enrichment by Western blot. Analyses of the subcellular fractions showed enrichment of WT, L168F, and W246G ELOVL4 proteins with enrichment of calnexin, validating that the proteins are present in the ER membrane (Fig. 1B). Surprisingly, for the first time, we also detected the presence of WT, L168F, and W246G ELOVL4 proteins in the nuclear fraction (Fig. 1B). Interestingly, we observed a double-banding pattern of ELOVL4 in the membrane fraction in our Western blot results, but much less in the nuclear fraction. Previous studies have confirmed that the double-banding pattern of ELOVL4 is due to the posttranslational modification of N-glycosylation (6, 19). However, N-glycosylation is irrelevant to ELOVL4’s enzyme activity (6).

### W246G ELOVL4 mutant impairs biosynthesis of 34:5n3 and 36:5n3 VLC-PUFA but accumulates C32 VLC-PUFA

**Fig. 2:**
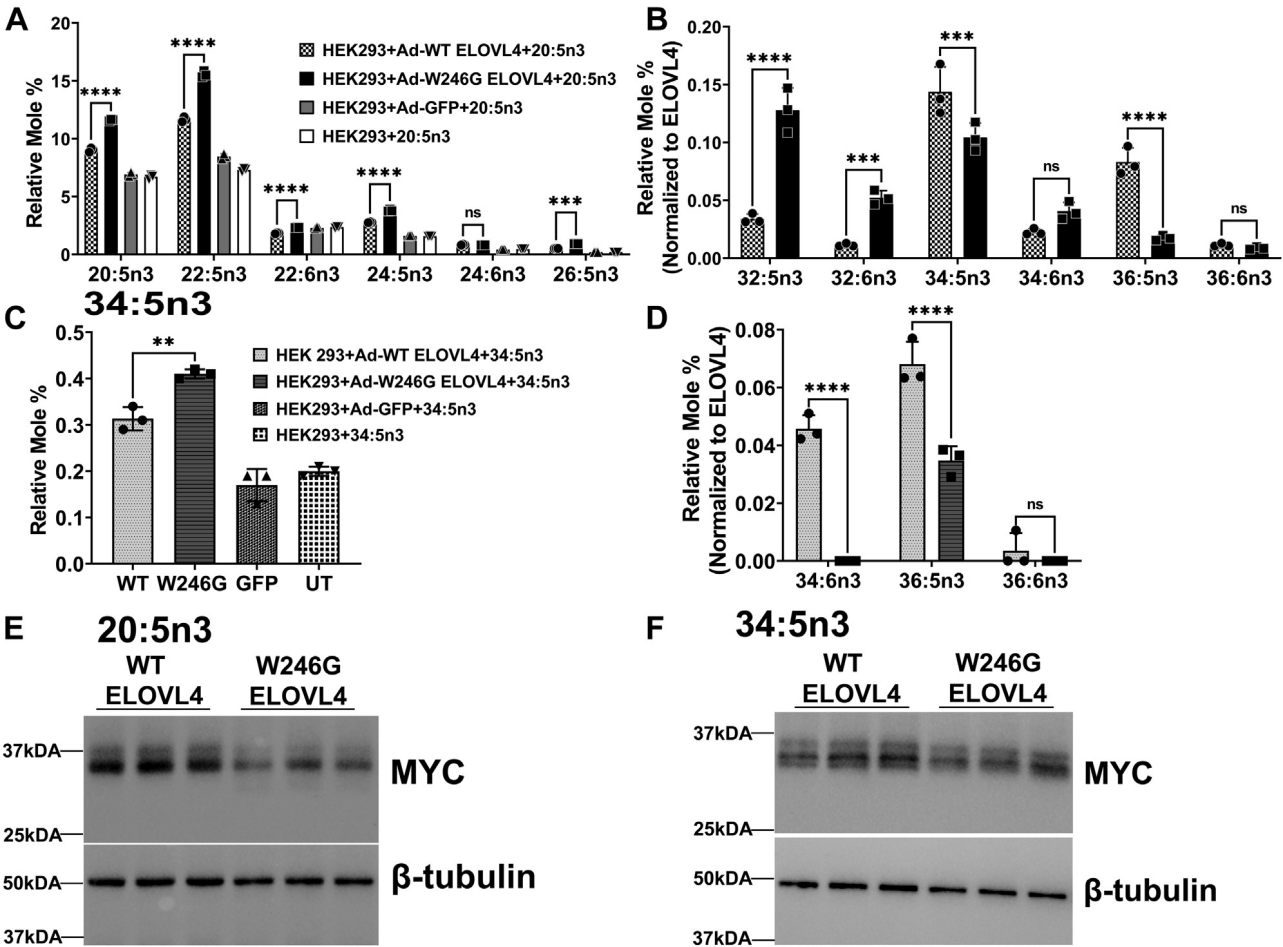
W246G ELOVL4 mutant impairs biosynthesis of 34:5n3 and 36:5n3 VLC-PUFA but accumulates C32 VLC-PUFA in HEK293 cells. A: Relative mole % of long-chain PUFA levels in HEK293 cells supplemented with 20:5n3 for 72 h in cells overexpressing W246G ELOVL4 mutant compared with WT ELOVL4 and control (GFP and untransduced [UT]) cells. Total lipids extracted from sample homogenate equivalent to 2.0 mg of protein were converted to FAMEs and analyzed by GC-FID. B: Relative mole % of VLC-PUFA levels normalized to WT ELOVL4 and W246G ELOVL4 protein expression levels in HEK293 cells after supplementation with 20:5n3.C: Uptake of 34:5n3 after 72 h in HEK293T cells overexpressing WT, W246G, and control cells (GFP and UT) supplemented with 34:5n3 VLC-PUFA. D: Elongated products of 34:5n3 VLC-PUFA normalized to WT ELOVL4 and W246G ELOVL4 protein expression. E: WT and W246G protein expression after 20:5n3 supplementation. Cell lysates were collected after 72 h for Western blot analysis with beta-tubulin as loading control. F: WT and W246G protein expression after 34:5n3 supplementation. Cell lysates were collected after 72 h for Western blot analysis with beta-tubulin as loading control. Results are the mean ± SD (n = 3). Statistical significance was assessed for A and C by ANOVA with Tukey’s post hoc test and B and D by ANOVA with Šídák’s post hoc test. ∗∗*P* < 0.01; ∗∗∗*P* < 0.001; ∗∗∗∗*P* < 0.0001, ns not significant in comparison with WT. ELOVL, elongation of very long-chain fatty acid; FAME, FA methyl ester; VLC-PUFA, very long chain-PUFA.

ELOVL4 is the only FA elongase (ELOVL) enzyme known to catalyze the biosynthesis of VLC-PUFA and VLC-SFA species with carbons ≥C28 (5, 19, 40). Patients carrying the W246G and L168F mutations have no reported retinal degeneration (30, 31). Since VLC-PUFA are predominantly enriched in the retina and are known to regulate photoreceptor health (9, 41), we analyzed the effect of the ELOVL4 mutants on VLC-PUFA synthesis. To explore the effect of the W246G mutation on VLC-PUFA biosynthesis, we expressed Myc-tagged *Elovl4* variants (WT and W246G) and GFP in HEK293 cells and confirmed protein expression. We chose HEK293 cells because we and others have shown in multiple studies that they possess the biosynthetic machinery to elongate FAs and synthesize VLC-FAs with the overexpression of the different ELOVLs and supplementation of VLC-FA precursors (5, 6, 19, 42). The transduced cells were supplemented with 20:5n3 (EPA), a precursor for VLC-PUFA biosynthesis. We showed that, compared with WT ELOVL4-expressing cells, W246G mutant ELOVL4-expressing cells had a significant uptake and accumulation of the supplemented 20:5n3 (Fig. 2A). At the same time, long-chain PUFA (LC-PUFA) metabolic derivatives formed from the elongation and desaturation of 20:5n3 FA, such as 22:5n3, 24:5n3, 26:5n3, and DHA (22:6n3), were also significantly higher in W246G-expressing cells than WT and controls (Fig. 2A). As expected, WT mouse ELOVL4 synthesized VLC-PUFA products, with 34:5n3 FA being the predominant VLC-PUFA followed by 36:5n3 FA (Fig. 2B). Unlike WT ELOVL4, W246G mutant ELOVL4 made significantly less 34:5n3 and 36:5n3 but significantly higher amounts of 32:5n3 and 32:6n3 VLC-PUFA (Fig. 2B). There were no VLC-PUFA products in either GFP or untransduced (UT) controls (Fig. 2B), indicating that the W246G mutant ELOVL4 is enzymatically active, in contrast to the 5-bp STGD3 mutant ELOVL4 that lacks VLC-PUFA biosynthesis (19). To further validate W246G protein’s deficiency in the biosynthesis of 34:5n3 and 36:5n3 VLC-PUFA when treated with 20:5n3, we supplemented HEK293 cells expressing the different ELOVL4 variants with 34:5n3 FA. Our FA analyses showed a significant accumulation of 34:5n3 in W246G-expressing cells compared with WT and controls (Fig. 2C). Although both the WT and W246G ELOVL4 successfully catalyzed the addition of two carbons to 34:5n3 to make 36:5n3, W246G ELOVL4-expressing cells made significantly less 36:5n3 (Fig. 2D). There were significant amounts of 34:6n3 in WT-expressing cells, which may indicate increased FADS2 desaturase activity on 34:5n3 or higher 22:6n3 elongation (Fig. 2D). Again, VLC-PUFA-elongated products of 34:5n3 were not detectable in the GFP and UT HEK cells. To account for differences in expression of the ELOVL4 protein levels that may affect the differences in the levels of synthesized VLC-PUFA, we normalized all levels of VLC-PUFA made to the level of WT and W246G ELOVL4 protein (Fig. 2E, F). Taken together, our data showed that the W246G mutation may alter the secondary structure of the protein in a way that reduces the biosynthesis of VLC-PUFA longer than 32 carbons.

### W246G SCA34 mutation makes significantly higher amounts of 32 carbon VLC-PUFA in the retina

**Fig. 3:**
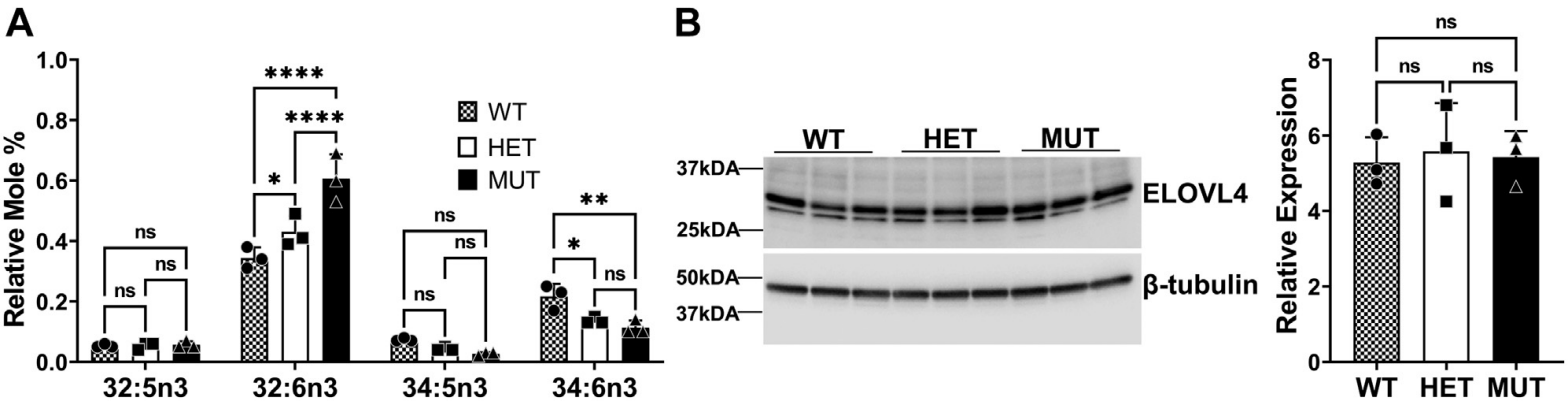
W246G mutant makes significantly higher amounts of 32 carbon VLC-PUFA in SCA34 homozygous mutant retinas. A: Relative mole % of VLC-PUFA levels in 12-month-old WT, heterozygous (HET), and homozygous (Mut) rat retinas with knock-in of the W246G mutation, analyzed by GC. B: ELOVL4 protein expression in 12-month-old WT, heterozygous (HET), and homozygous (Mut) rats with knock-in of the W246G mutation. Tissue lysates were collected for Western blot analysis with beta-tubulin as loading control. Results are the mean ± SD (n = 3). Statistical significance was assessed by ANOVA with Tukey’s post hoc test. ∗*P* < 0.05; ∗∗*P* < 0.01, ns not significant in comparison with WT. ELOVL, elongation of very long-chain fatty acid; SCA, spinocerebrellar ataxia; VLC-PUFA, very long chain-PUFA.

We generated and characterized a Long Evans rat model of SCA34 (14, 38) in which we knocked in the c.736T > G (p. W246G) mutation that causes SCA34 (31). Our behavioral and functional analyses revealed that homozygous W246G SCA34 rats (MUT) develop impaired synaptic plasticity, skin lesions, and reduced a- and b-wave electroretinography amplitudes without any retinal degeneration (14, 38). Our lipid analysis of the SCA34 rats showed significant deficiency in skin VLC-SFA levels; interestingly, there were no significant differences in the levels of total retinal VLC-PUFA in the WT, HET, and MUT rats (38). Whether W246G mutation alters the biosynthesis of individual VLC-PUFA species in the retina of our SCA34 W246G KI animal model was not explored. To validate our in vitro results that W246G ELOVL4 mutant impairs biosynthesis of 34:5n3 and 36:5n3 VLC-PUFA, but accumulates C32 VLC-PUFA in vivo, we extracted and analyzed FAs from retinas of WT, HET, and MUT SCA34 rats. Our results from the 12-month-old rats showed that 32:6n3 FA was significantly higher in MUT rat retinas than in their WT and HET littermates (Fig. 3A). There were significantly lower amounts of 34:6n3 in MUT retinas than in WT and HET retinas. The level of 34:5n3 in MUT retinas was lower than in WT and HET retinas, although not significantly different across the genotypes. The expression levels of ELOVL4 across all genotypes at 12 months were not significantly different (Fig. 3B), indicating that differences in VLC-PUFA levels are primarily due to the W246G mutation altering VLC-PUFA synthesis and not due to levels of protein expression.

### L168F ELOVL4 mutant enhances biosynthesis of VLC-PUFA with carbon chains greater than C34, unlike W246G mutant ELOVL4

**Fig. 4:**
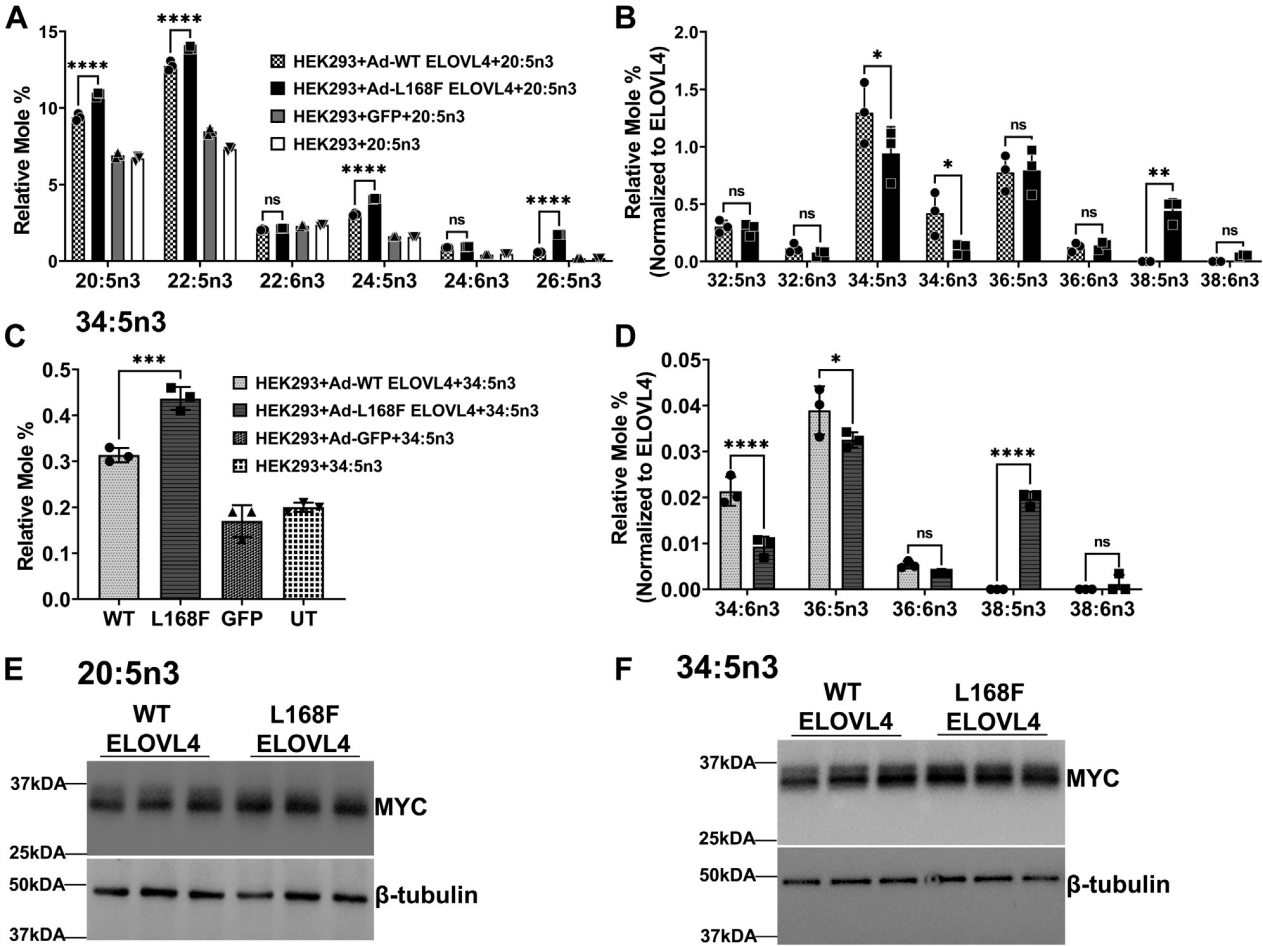
L168F ELOVL4 mutant enhances biosynthesis of VLC-PUFA with carbon chains greater than C34. A: Long-chain PUFA levels in HEK293 cells supplemented with 20:5n3 for 72 h in cells overexpressing L168F ELOVL4 compared with WT ELOVL4 and control (GFP and untransduced [UT]) cells. B: Relative mole % of VLC-PUFA levels normalized to WT ELOVL4 and L168F ELOVL4 protein levels after 20:5n3 supplementation. C: Uptake of 34:5n3 after 72 h in HEK293T cells overexpressing WT, L168F, and control cells (GFP and UT) supplemented with 34:5n3. D: Elongated products of 34:5n3 normalized to WT ELOVL4 and L168F ELOVL4 expression levels. E: WT and L168F ELOVL4 protein expression after 20:5n3 supplementation. Cell lysates were collected after 72 h for Western blot analysis with beta-tubulin as loading control. F: WT and L168F ELOVL4 protein expression after 34:5n3 supplementation. Cell lysates were collected after 72 h for Western blot analysis with beta-tubulin as loading control. Results are the mean ± SD (n = 3). Statistical significance was assessed for A and C by ANOVA with Tukey’s post hoc test and B and D by ANOVA with Šídák’s post hoc test. ∗*P* < 0.05; ∗∗*P* < 0.01; ∗∗∗*P* < 0.001; ∗∗∗∗*P* < 0.0001, ns not significant in comparison with WT. ELOVL, elongation of very long-chain fatty acid; UT, untransduced; VLC-PUFA, very long chain-PUFA.

Since patients with L168F ELOVL4 mutation do not have any reported retinal degeneration (30), we determined the effect of the L168F mutation on VLC-PUFA biosynthesis in HEK293 cells transduced with Myc-tagged *Elovl4* variants (WT and L168F) or GFP and UT control cells supplemented with 20:5n3. Similar to W246G-expressing cells, there was significant accumulation of the supplemented 20:5n3 compared with WT ELOVL4 and controls (Fig. 4A). LC-PUFA metabolic intermediates from supplemented 20:5n3, such as 22:5n3, 24:5n3, and 26:5n3, were significantly higher in L168F-expressing cells than in WT-expressing cells and controls (Fig. 4A). However, WT ELOVL4 and L168F synthesized similar amounts of 32:5n3, 32:6n3, 36:5n3, and 36:6n3 (Fig. 4B). Similar to WT ELOVL4-expressing cells, 34:5n3 was the predominant VLC-PUFA made by the L168F mutant ELOVL4, although in a significantly lower amount (Fig. 4B). WT ELOVL4 also synthesized significantly higher amounts of 34:6n3 than did the L168F mutant (Fig. 4B). Interestingly, the L168F mutant ELOVL4 further elongated 36:5n3 to 38:5n3, which was not detectable in WT ELOVL4-expressing cells (Fig. 4B).

Despite the ability of the L168F mutant ELOVL4 to synthesize VLC-PUFA, we showed that the L168F-transduced cells supplemented with 34:5n3 (Fig. 4C) made significantly lower levels of 34:6n3 and 36:5n3 than WT-transduced cells (Fig. 4D). However, the L168F-expressing cells elongated the supplemented 34:5n3 to 38:5n3, which was again not detected in WT ELOVL4-expressing cells (Fig. 4C). We normalized the levels of VLC-PUFA synthesized to the level of ELOVL4 protein expressed, which was not significantly different in the 20:5n3- and 34:5n3-supplemented WT and L168F-expressing cells (Fig. 4D–E). Taken together, our data show that L168F mutant ELOVL4 may alter the secondary structure of the ELOVL4 protein in a way that further enhances the elongation of VLC-PUFA beyond 34 carbons in length.

### W246G, L168F, and STGD3 (5-bp deletion) mutations suppress VLC-SFA biosynthesis

**Fig. 5:**
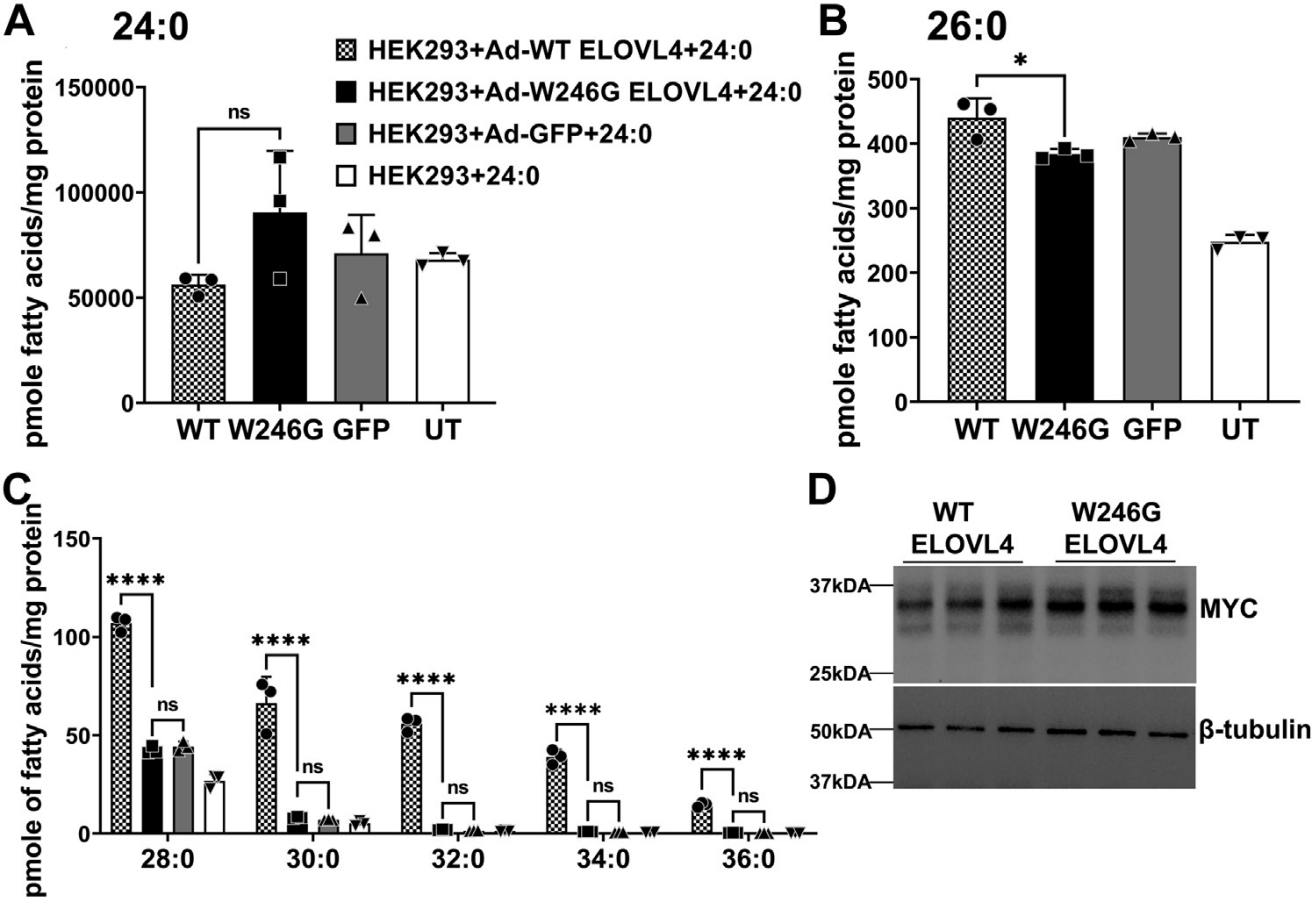
W246G is deficient in very long chain saturated FA synthesis. A: Uptake of 24:0 after 72 h in HEK293 cells overexpressing WT, W246G, and control cells (GFP and UT) supplemented with 24:0 SFA. B: Total 26:0, an elongation product of 24:0, in the cells supplemented with 24:0 SFA. C: VLC-SFA levels in cells described in A. VLC-SFAs in W246G-expressing cells were not significantly different from GFP control cells and were therefore not normalized to WT ELOVL4 and W246G ELOVL4 protein expression. D: WT and W246G ELOVL4 protein expression after 24:0 supplementation. Cell lysates were collected after 72 h for Western blot analysis with beta-tubulin as loading control. Results are the mean ± SD (n = 3). Statistical significance was assessed for A, B, and C by ANOVA with Tukey’s post hoc test. ∗*P* < 0.05; ∗∗∗∗*P* < 0.0001, ns not significant in comparison with WT. ELOVL, elongation of very long-chain fatty acid; UT, untransduced; VLC-SFA, very long chain-saturated FA.

The ELOVL4 enzyme is not only able to elongate LC-PUFA but also mediates the elongation of long-chain saturated FAs into VLC-SFA, which are necessary for brain and skin function (5, 17, 19, 40). Not only does the W246G and L168F mutation cause SCA34, but some patients carrying the L168F mutation also develop EKV (30). Meanwhile, patients carrying the STGD3 (5-bp deletion) mutation have no reported SCA and EKV, and previous cell culture studies indicate that the STGD3 mutant is enzymatically inactive in the biosynthesis of VLC-PUFA. Therefore, we investigated the effect of the W246G, L168F, and STGD3 mutation on VLC-SFA biosynthesis in vitro. We expressed Myc-tagged *Elovl4* variants (WT, W246G, and L168F) and HA-tagged *Elovl4* variants (WT and STGD3) or GFP in HEK293 cells and supplemented them with the VLC-SFA precursor 24:0. Following the supplementation of 24:0, W246G-overexpressing cells had significantly lower amounts of 26:0 than did WT ELOVL4-expressing cells (Fig. 5A, B). As expected, WT ELOVL4 elongated the 26:0 substrates by adding a series of two carbons to make 28:0, 30:0, 32:0, 34:0, and 36:0 (Fig. 5C). However, the W246G ELOVL4 did not make significant amounts of VLC-SFAs compared with controls, indicating a deficiency in the W246G ELOVL4 in elongating LC-SFAs. ELOVL4 expression in WT and W246G-expressing cells was not significantly different (Fig. 5D).

**Fig. 6:**
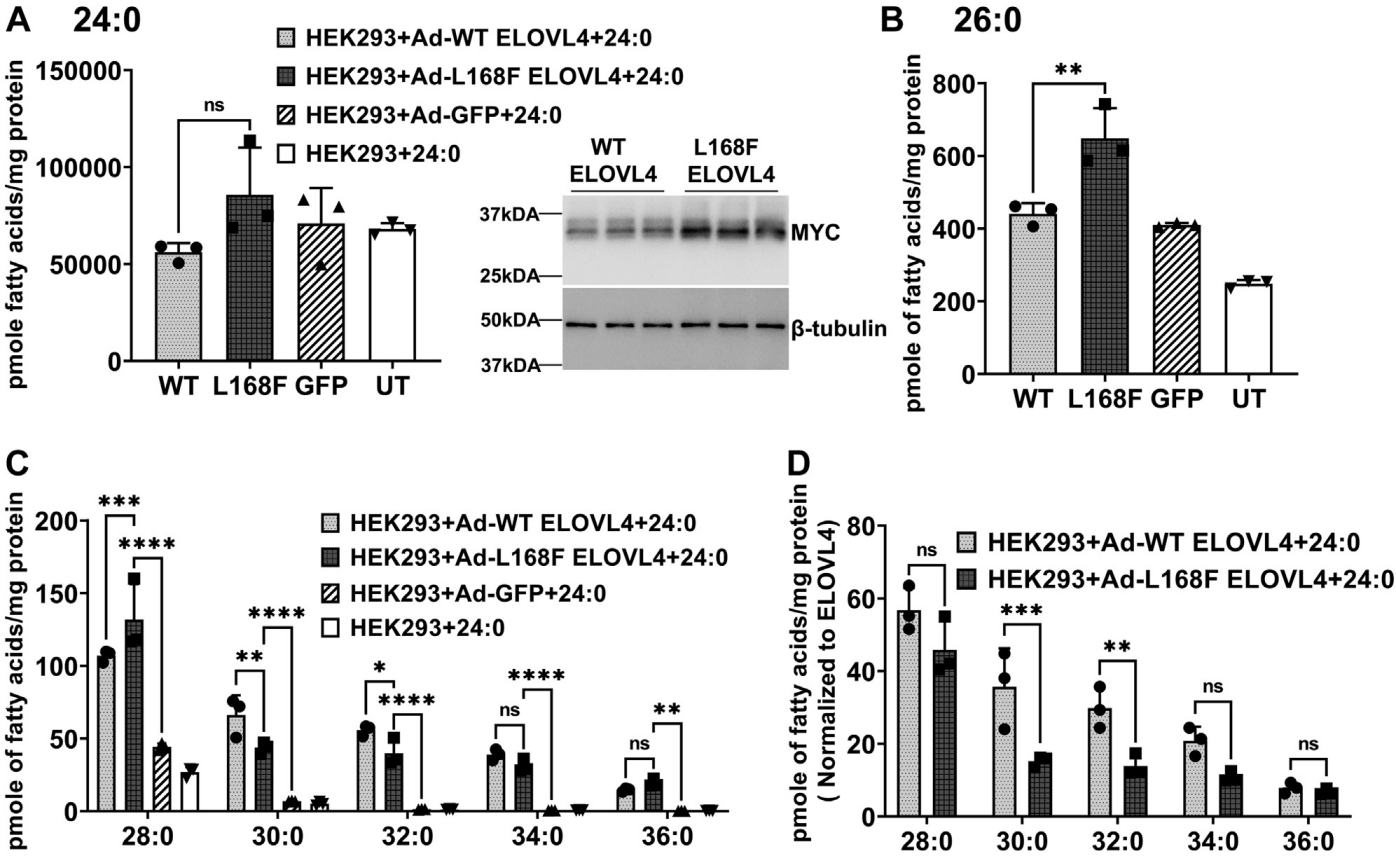
L168F SCA34 mutant suppresses very long chain saturated FA synthesis. A: Uptake of 24:0 after 72 h in HEK293 cells overexpressing WT, L168F, and control cells (GFP and UT) supplemented with 24:0 SFA. B: Total 26:0 in the cells supplemented with 24:0 SFA. C: Levels of VLC-SFAs after 24:0 supplementation. L168F-overexpressing cells had significant levels of VLC-SFAs compared with GFP controls. Western blot showing WT, L168F ELOVL4, and GFP protein expression after 24:0 supplementation. Cell lysates were collected after 72 h for Western blot analysis with beta-tubulin as loading control. D: VLC-SFA levels normalized to WT ELOVL4 and L168F ELOVL4 protein expression in cells after treatment with 24:0 FA. VLC-SFAs synthesized by L168F ELOVL4 (30:0 and 32:0) were significantly lower than WT levels. Results are the mean ± SD (n = 3). Statistical significance was assessed for A, B, and C by ANOVA with Tukey’s post hoc test and D by ANOVA with Šídák’s post hoc test. ∗*P* < 0.05; ∗∗*P* < 0.01; ∗∗∗*P* < 0.001; ∗∗∗∗*P* < 0.0001, ns not significant in comparison with WT. ELOVL, elongation of very long-chain fatty acid; SCA, spinocerebrellar ataxia; UT, untransduced; VLC-SFA, very long chain-saturated FA.

**Fig. 7:**
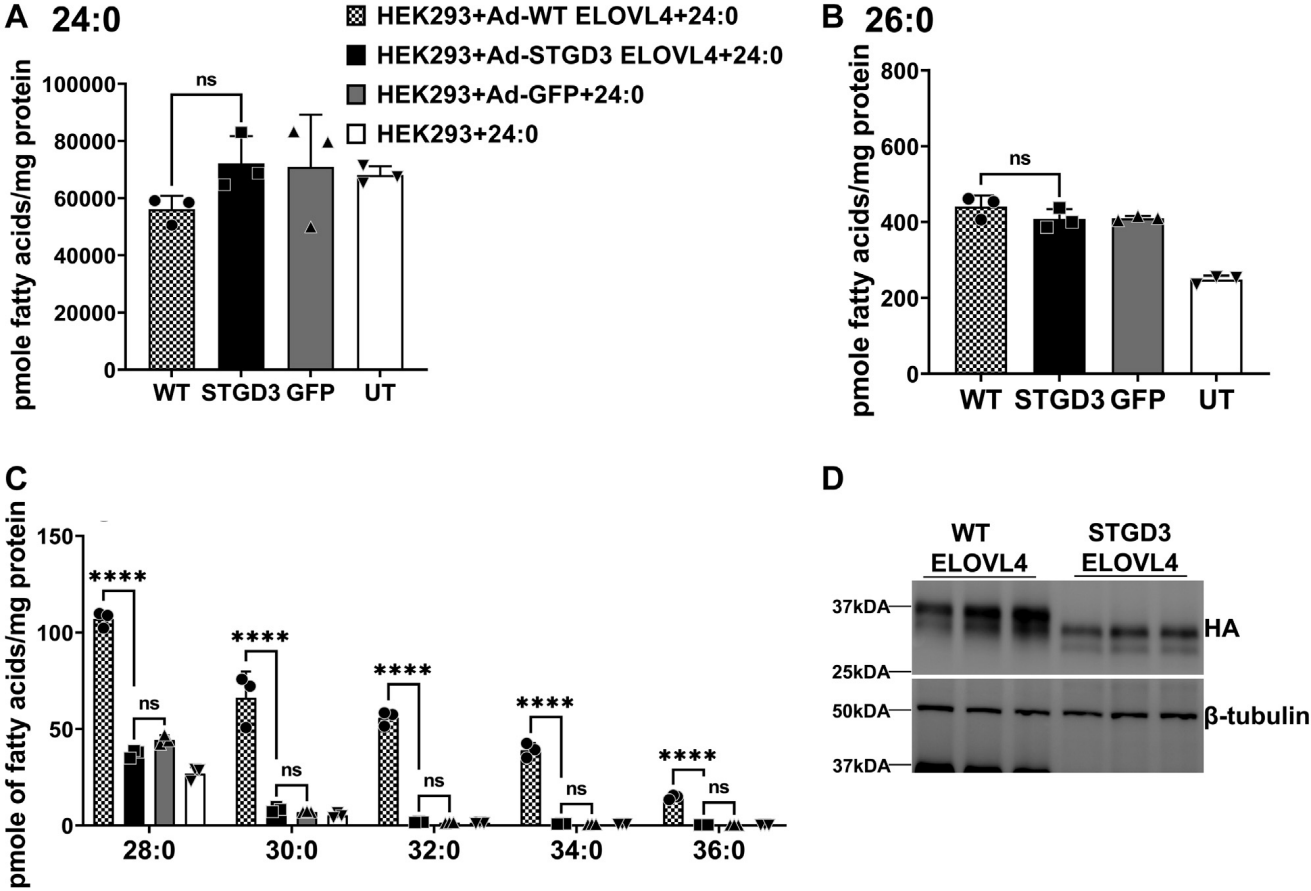
STGD3 is deficient in very long chain saturated FA synthesis. A: Uptake of 24:0 after 72 h in HEK293 cells overexpressing WT, STGD3, and control cells (GFP and UT) supplemented with 24:0 SFA. B: Total 26:0 in the cells supplemented with 24:0 SFA. C: VLC-SFA levels in cells described in A. VLC-SFAs in STGD3-expressing cells were not significantly different from GFP control cells and were therefore not normalized to WT ELOVL4 and W246G ELOVL4 protein expression. D: WT and STGD3 ELOVL4 protein expression after 24:0 supplementation. Cell lysates were collected after 72 h for Western blot analysis with beta-tubulin as loading control. Results are the mean ± SD (n = 3). Statistical significance was assessed for A, B, and C by ANOVA with Tukey’s post hoc test. ∗∗∗∗*P* < 0.0001, ns not significant in comparison with WT. ELOVL, elongation of very long-chain fatty acid; VLC-SFA, very long chain-saturated FA; STGD3, Autosomal Stargardt-like Macular Dystrophy; UT, untransduced.

Unlike the W246G mutant ELOVL4-expressing cells that have lower levels of 26:0, there was a significant accumulation of 26:0 in L168F-expressing cells compared with WT and control cells following the supplementation of 24:0 (Fig. 6A, B). All of the VLC-SFAs, including 28:0, 30:0, 32:0, 34:0, and 36:0 synthesized from the elongation of 26:0 by L168F ELOVL4, were significantly higher than in GFP controls (Fig. 6C). However, 30:0 and 32:0 were significantly lower in L168F-transduced cells when the VLC-SFAs were normalized to WT ELOVL4 and L168F ELOVL4 protein expression (Fig. 6D). While the levels of 24:0 and 26:0 SFA were similar in WT ELOVL4-expressing cells and STGD3 (Fig. 7A-B), the STGD3 mutant protein did not make a significant amount of VLC-SFAs compared with GFP controls (Fig. 7C) after supplementation of 24:0 and were therefore not normalized to STGD3 ELOVL4 protein expression (Fig. 7D).

**Fig. 8:**
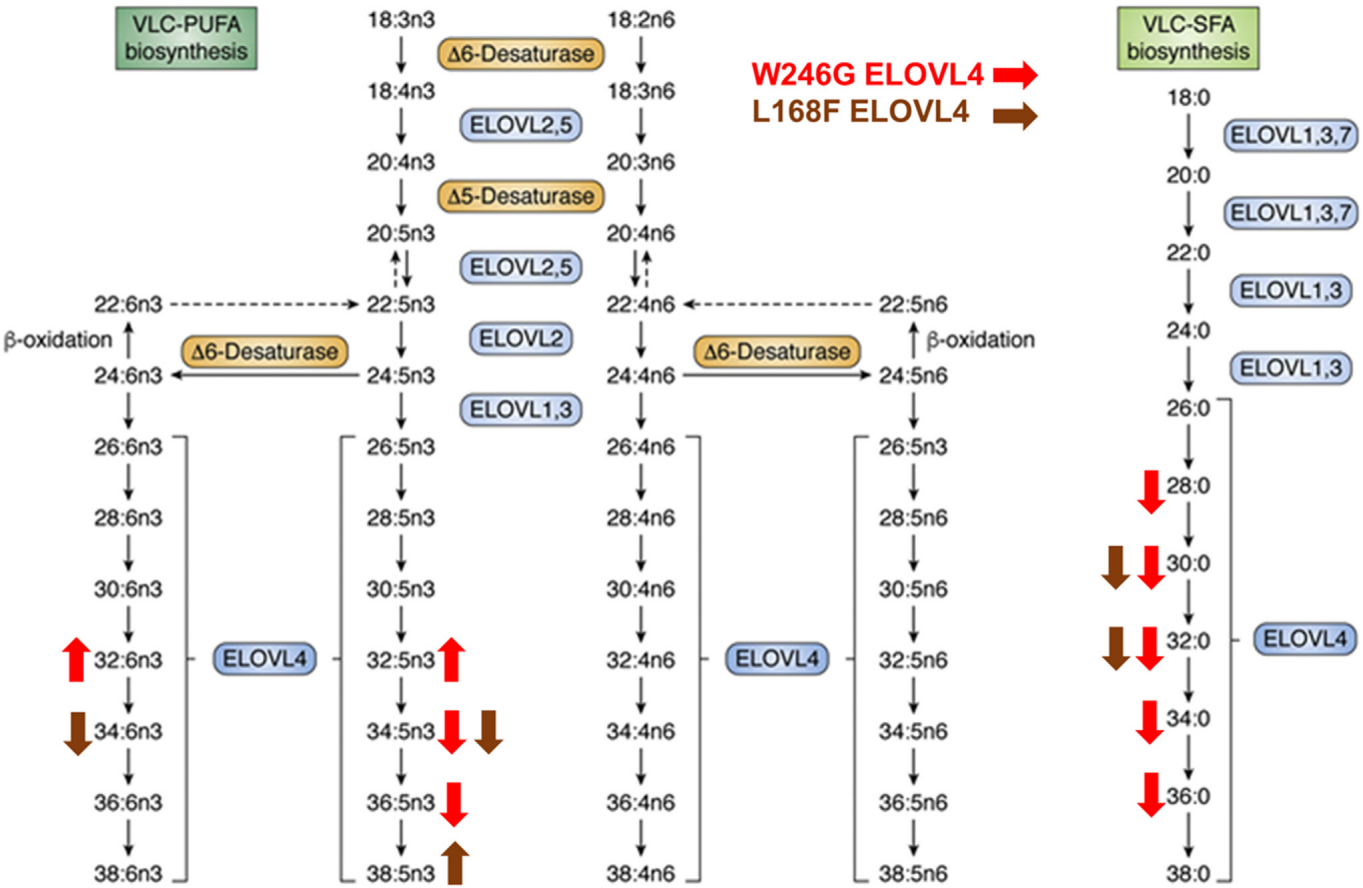
VLC-SFA and VLC-PUFA elongation pathways. VLC-PUFAs result from a series of elongation steps catalyzed by ELOVL 1, 2, 3, 4, and 5 and desaturation steps catalyzed by Δ5 Desaturase (FADS1) and Δ6 desaturase (FADS2). VLC-SFA biosynthesis requires the expression of combinations of ELOVL1, 3, 6, 4, and 7. W246G and L168F ELOVL4 mutations differentially alter VLC-FA synthesis. W246G ELOVL4 leads to the accumulation of 32:6n3 and 32:5n3 but leads to decreases in 34:5n3, 34:6n3, and 36:5n3. L168F mutant ELOVL4 decreases 34:6n3 and 34:5n3 VLC-PUFA but enhances the synthesis of 38:5n3. L168F mutation decreases 30:0 and 32:0, whereas W246G ELOVL4 shows negligible activity in the biosynthesis of 28:0, 30:0, 32:0, 34:0, and 36:0 in vitro. This figure is an adapted reproduction from Gyening Kofi Yeboah *et al.* (2021). J. Lipid Res. https://doi.org/10.1016/j.jlr.2021.100030. ELOVL, elongation of very long-chain fatty acid; VLC-SFA, very long chain-saturated FA; VLC-PUFA, very long chain-PUFA.

These results indicate that, unlike the W246G and STGD3 ELOVL4 that lacks the ability to synthesize VLC-SFAs, L168F ELOVL4 has a better efficiency towards elongation of LC-SFA to make VLC-SFA, although to a lesser extent than WT ELOVL4 (Fig. 8).

## Discussion

FA elongation occurs through a four-step process of condensation, reduction, dehydration, and reduction, with two carbon atoms added to existing FA substrates. ELOVL1-7 catalyzes the first and rate-limiting condensation reaction between a fatty acyl-CoA and malonyl-CoA. FA substrates for VLC-SFA biosynthesis require the expression of combinations of ELOVL 1, 3, 6, and 7. Meanwhile, VLC-PUFAs result from a series of elongation steps catalyzed by ELOVL 1, 2, 3, 4, 5 and desaturation steps catalyzed by Δ5 Desaturase (FADS1) and Δ6 desaturase (FADS2). The ELOVL4 enzyme is expressed in only a few tissues in which it differentially synthesizes both VLC-SFA and VLC-PUFA (5, 6, 19, 43). In the skin (8, 17, 24, 25), brain (7), and Meibomian glands (13, 44), the products are mainly VLC-SFA, while in the retina (20, 45) and testes (46, 47, 48, 49) the main products are VLC-PUFA. Four mutations in exon 6 of *ELOVL4* cause Stargardt-like Macular Dystrophy (STGD3), a juvenile form of macular degeneration (26, 27, 28, 29, 50), while five point mutations in exons 4 and 6 cause SCA34 (30, 31, 32, 33, 51), a motor impairment caused by degeneration of neurons in the cerebellum. Some of the SCA34 patients also develop EKV. Interestingly, and germane to this study, although the mutations are in the same gene, STGD3 patients have no reported CNS or skin phenotype and SCA34 patients have no reported macular degeneration. However, how these different ELOVL4 mutations cause such diverse tissue-specific disorders is not completely understood.

In the present study, we determined how two different ELOVL4 mutations (W246G and L168F) affect the quantity of VLC-FA biosynthesis in vitro*.* Our results showed that, unlike the truncated STGD3-causing mutant ELOVL4 that lacks ER targeting/retention motif and is mislocalized away from the ER (6, 52, 53), the WT ELOVL4, L168F, and W246G mutants localize to ER membranes, which is necessary for its function (6). Surprisingly, for the first time, we detected the presence of ELOVL4 in nuclear fractions prepared from subcellular fractionation of the cells expressing the different ELOVL4 variants. Since the nuclear membrane is continuous with the ER membrane (54), this may be an explanation for the presence of ELOVL4 protein in the nuclear fraction. However, we do not rule out the potential that ELOVL4 plays a role in the nucleus, which may be physiologically important in ELOVL4-expressing tissues (6, 19).

We previously showed that the MUT Long Evans rat model of the W246G SCA34 pathology develops age-related motor deficits, impaired synaptic plasticity, retinal dysfunction, and EKV (14, 38). Even though total levels of retinal VLC-PUFA in WT, HET, and MUT rats were comparable, skin VLC-SFA levels were decreased in HET and MUT rats compared with WT rats (38). Our previous studies, however, did not investigate how the biosynthesis of individual VLC-PUFA species was impacted. The present study explored individual VLC-PUFA biosynthesis by the W246G and L168F mutant ELOVL4 variants in vitro. Our FA analyses showed that the W246G had impaired biosynthesis of 34:5n3 and 36:5n3 but increased synthesis of 32:5n3 and 32:6n3 (Fig. 2B). Considering the changes we observed in retina function in the MUT W246G rats, we determined and confirmed alterations in retinal VLC-PUFA levels. In the retinas of the SCA34 rats, we observed accumulation of 32:6n3 (Fig. 3A), which is consistent with the in vitro VLC-PUFA biosynthetic data (Fig. 2B).

On the other hand, in vitro L168F ELOVL4 makes equivalent amounts of C32 VLC-PUFA products similar to the WT ELOVL4. Strikingly, the L168F mutant ELOVL4 seems to have enhanced elongase activity towards biosynthesis of VLC-PUFA with carbon chains greater than C34. The L168F mutant ELOVL4 elongated and made both 38:5n3 and 38:6n3, which were not detectable in WT ELOVL4-overexpressing cells. Thus, it is clear that, although W246G and L168F mutant ELOVL4 variants have VLC-PUFA biosynthetic activity, the individual FA that each of these different mutant proteins synthesizes is different (Fig. 8).

Consistent with the SCA34 rats, in which we showed significantly reduced VLC-SFA in the skin (38), our in vitro results indicate that the W246G mutant ELOVL4 is significantly deficient in VLC-SFA biosynthesis. L168F ELOVL4 is also significantly deficient in 30:0 and 32:0 VLC-SFA biosynthesis, although less severe than that observed with the W246G ELOVL4. These mutations likely alter the structure of the protein to selectively stymie the initial rate-limiting condensation reactions that result in the synthesis of VLC-SFA. Based on our data and others, the nontransduced HEK293T cells are unable to produce any detectable VLC-PUFA with or without the supplementation of EPA. However, background levels of VLC-SFA exist with or without supplementation of 24:0 (5, 6, 19, 43).

Although the structure of ELOVL4 has not been elucidated, bioinformatic tools have predicted ELOVL4 as well as Sur4p/Elo3p, the yeast homolog of ELOVL4, to be 7-pass transmembrane proteins (31). Furthermore, crystallographic studies of ELOVL7 show that the protein has 7-transmembrane helices and that the amino acids that line the transmembrane 6 (TM6) and TM7 loop, as well as the catalytic region, are most likely to determine the size and shape of the FA-acyl substrate binding and catalytic region (55). An alteration in the size and shape of the substrate and catalytic region restricts entry of the substrates and the length of products produced (55). Thus, the L168F and W246G SCA34-causing mutations may alter the length and shape of the catalytic region, especially since the substitution of phenylalanine for leucine is not far from the substrate binding and catalytic region and the substitution of glycine for tryptophan in W246G is within the proposed critical N-terminal region of TM7 between the TM6 and TM7 loop (31, 56). Such potential modifications in the size and shape of the catalytic and substrate-binding region could alter the protein substrate preferences leading to the changes in quantity and type of VLC-FA products measured in our experiments. Future crystallographic studies of the mutant enzymes will be needed to resolve these questions.

Taken together, we have demonstrated that, consistent with the different tissue-specific disorders caused by different ELOVL4 mutations, W246G and L168F SCA34 ELOVL4 mutations have some VLC-PUFA biosynthetic ability. The ability of these mutants to make some levels of VLC-PUFAs, which may be sufficient to preserve retinal structure (9, 17, 18), may explain why SCA34 patients do not have any reported retinal degeneration compared to STGD3 patients in which the STGD3 mutant ELOVL4 lacks VLC-PUFA biosynthesis. However, the W246G and L168F mutations appear to be deficient in biosynthesis of VLC-SFA products necessary for neurotransmission and skin barrier function (7, 8, 17), which may explain why these patients develop cerebellar neuron degenerations that lead to progressive neuromuscular deficits as well as EKV. Our future studies will continue to investigate the factors that control tissue-specific biosynthesis of these FAs and determine if dietary supplementation of these FAs will attenuate progression of some of these tissue-specific disorders.

## Data Availability

All data are contained within this manuscript.

## Conflict of Interest

The authors declare no conflict of interest. Martin-Paul Agbaga and Richard S. Brush have United States Patent 8,021,874 B2 issued on September 20, 2011 covering Very long chain polyunsaturated FAs, methods of production, and uses.
